# 2-(4-*tert*-Butyl­phen­yl)-5-*p*-tolyl-1,3,4-oxadiazole

**DOI:** 10.1107/S1600536809051198

**Published:** 2009-12-04

**Authors:** Biao Jin

**Affiliations:** a School of Chemistry and Chemical Engineering, Southeast University, Nanjing 210096, People’s Republic of China

## Abstract

In the title compound, C_19_H_20_N_2_O, the dihedral angles between the 1,3,4-oxadiazole ring and the pendant 4-*tert*-butyl­phenyl and 4-methyl­phenyl rings are 12.53 (17) and 2.14 (17)°, respectively. In the crystal, mol­ecules are linked by C—H⋯N hydrogen bonds, forming chains.

## Related literature

For background to the applications of 1,3,4-oxadiazo­les, see: Jin *et al.* (2004 ▶).
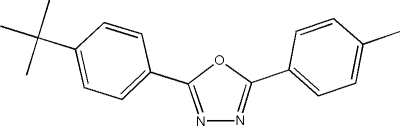


## Experimental

### 

#### Crystal data

C_19_H_20_N_2_O
*M* *_r_* = 292.37
Monoclinic, 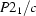

*a* = 9.886 (9) Å
*b* = 10.613 (9) Å
*c* = 16.093 (13) Å
β = 99.14 (2)°
*V* = 1667 (2) Å^3^
*Z* = 4
Mo *K*α radiation
μ = 0.07 mm^−1^
*T* = 298 K
0.20 × 0.20 × 0.20 mm

#### Data collection

Rigaku SCXmini diffractometer
Absorption correction: multi-scan (*CrystalClear*; Rigaku, 2005 ▶) *T*_min_ = 0.813, *T*_max_ = 1.000
17627 measured reflections
3791 independent reflections
3032 reflections with *I* > 2σ(*I*)
*R*_int_ = 0.063

#### Refinement

*R*[*F* ^2^ > 2σ(*F* ^2^)] = 0.109
*wR*(*F* ^2^) = 0.223
*S* = 1.15
3791 reflections
199 parameters
H-atom parameters constrained
Δρ_max_ = 0.31 e Å^−3^
Δρ_min_ = −0.27 e Å^−3^

### 

Data collection: *CrystalClear* (Rigaku, 2005 ▶); cell refinement: *CrystalClear*; data reduction: *CrystalClear*; program(s) used to solve structure: *SHELXS97* (Sheldrick, 2008 ▶); program(s) used to refine structure: *SHELXL97* (Sheldrick, 2008 ▶); molecular graphics: *SHELXTL* (Sheldrick, 2008 ▶); software used to prepare material for publication: *PRPKAPPA* (Ferguson, 1999 ▶).

## Supplementary Material

Crystal structure: contains datablocks I, global. DOI: 10.1107/S1600536809051198/hb5237sup1.cif

Structure factors: contains datablocks I. DOI: 10.1107/S1600536809051198/hb5237Isup2.hkl

Additional supplementary materials:  crystallographic information; 3D view; checkCIF report

## Figures and Tables

**Table 1:** Hydrogen-bond geometry (Å, °)

*D*—H⋯*A*	*D*—H	H⋯*A*	*D*⋯*A*	*D*—H⋯*A*
C10—H10*A*⋯N2^i^	0.93	2.59	3.456 (5)	155

Symmetry code: (i) 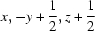.

**Fig. 1.**
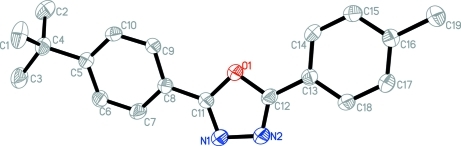
The molecular structure of (I) with displacement ellipsoids drawn at the 30% probability level (H atoms have been omitted for clarity).

**Fig. 2.**
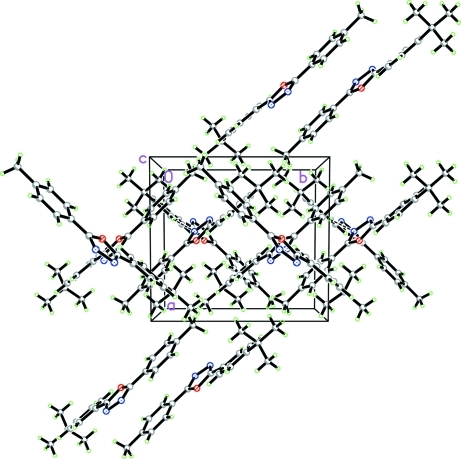
The packing of (I).

**Fig. 3.**
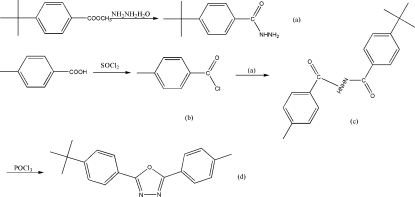
The formation of (I).

## supplementary crystallographic information

### Experimental

To a 100 ml three-neck flask, 4-*tert*-Butylbenzoic acid methyl ester (19.2 g, 0.1 mol) and 30 ml ethanol were added, and heated to reflux, then hydronium (85%, 6 g, 0.1 mol) was dropped. The mixture was stirred at 353 K for 7 h under nitrogen. After finishing the reaction, the mixture was cooled to the room temperature, and dumped into ten folds of excess water to afford plenty of white solid, filtered and washed with water to yield 4-*tert*-butylbenzhydrazide (*a*) (15.6 g, yield = 81%).

4-Methyl benzoic acid (6.8 g, 0.1 mol)was added into a 50 ml three-neck flask, then heated to 313 K, and 10 ml (excessive) thionyl chloride was dropped slowly. The mixture was heated to 353 K, and refluxed for 7 h. After finishing the reaction, distilling residual thionyl chloride with rotary evaporator, getting slightly yellow liquid 4-methylbenzoyl chloride (*b*).

To a 500 ml single-neck flask, 15.6 g (0.08 mol, excessive) (*a*), 250 ml tetrahydrofuran, 7.7 g (0.05 mol) (*b*) and 1 ml pyridine were added. The mixture was refluxed for 12 h. After finishing the reaction, the residual tetrahydrofuran was distilled and the mixture was precipitated into ice-water, filtered and washed with water to afford white solid. The crude product was recrystallized with ethanol three times to yield white sheet crystal (*c*) (13.4 g, yield 84%). A mixture of (*c*) (13.4 g, 42.2 mmol) and POCl_3_ (250 ml) was stirred at 378 K for about 10 h, then poured into ice-water to yield slightly yellow crystals. The mixture was recrystallized with ethanol three times to afford colourless prisms of (I).

### Refinement

Positional parameters of all the H atoms were calculated geometrically and were allowed to ride on the C atoms to which they are bonded, with *U*_iso_(H) = 1.2U_eq_(C).

### Crystal data

**Table:**

C_19_H_20_N_2_O	*F*(000) = 624
*M**_r_* = 292.37	*D*_x_ = 1.165 Mg m^−^^3^
Monoclinic, *P*2_1_/*c*	Mo *K*α radiation, λ = 0.71073 Å
Hall symbol: -P 2ybc	Cell parameters from 3230 reflections
*a* = 9.886 (9) Å	θ = 2.8–27.4°
*b* = 10.613 (9) Å	µ = 0.07 mm^−^^1^
*c* = 16.093 (13) Å	*T* = 298 K
β = 99.14 (2)°	Prism, colourless
*V* = 1667 (2) Å^3^	0.20 × 0.20 × 0.20 mm
*Z* = 4	

### Data collection

**Table:**

Rigaku SCXmini diffractometer	3791 independent reflections
Radiation source: fine-focus sealed tube	3032 reflections with *I* > 2σ(*I*)
graphite	*R*_int_ = 0.063
Detector resolution: 13.6612 pixels mm^-1^	θ_max_ = 27.4°, θ_min_ = 3.2°
ω scans	*h* = −12→12
Absorption correction: multi-scan (*CrystalClear*; Rigaku, 2005)	*k* = −13→13
*T*_min_ = 0.813, *T*_max_ = 1.000	*l* = −20→20
17627 measured reflections	

### Refinement

**Table:**

Refinement on *F*^2^	Primary atom site location: structure-invariant direct methods
Least-squares matrix: full	Secondary atom site location: difference Fourier map
*R*[*F*^2^ > 2σ(*F*^2^)] = 0.109	Hydrogen site location: inferred from neighbouring sites
*wR*(*F*^2^) = 0.223	H-atom parameters constrained
*S* = 1.15	*w* = 1/[σ^2^(*F*_o_^2^) + (0.0626*P*)^2^ + 1.4014*P*] where *P* = (*F*_o_^2^ + 2*F*_c_^2^)/3
3791 reflections	(Δ/σ)_max_ < 0.001
199 parameters	Δρ_max_ = 0.31 e Å^−^^3^
0 restraints	Δρ_min_ = −0.27 e Å^−^^3^

### Special details

**Table:**

Geometry. All e.s.d.'s (except the e.s.d. in the dihedral angle between two l.s. planes) are estimated using the full covariance matrix. The cell e.s.d.'s are taken into account individually in the estimation of e.s.d.'s in distances, angles and torsion angles; correlations between e.s.d.'s in cell parameters are only used when they are defined by crystal symmetry. An approximate (isotropic) treatment of cell e.s.d.'s is used for estimating e.s.d.'s involving l.s. planes.
Refinement. Refinement of *F*^2^ against ALL reflections. The weighted *R*-factor *wR* and goodness of fit *S* are based on *F*^2^, conventional *R*-factors *R* are based on *F*, with *F* set to zero for negative *F*^2^. The threshold expression of *F*^2^ > σ(*F*^2^) is used only for calculating *R*-factors(gt) *etc*. and is not relevant to the choice of reflections for refinement. *R*-factors based on *F*^2^ are statistically about twice as large as those based on *F*, and *R*- factors based on ALL data will be even larger.

### Fractional atomic coordinates and isotropic or equivalent isotropic displacement parameters (Å^2^)

**Table:**

	*x*	*y*	*z*	*U*_iso_*/*U*_eq_	
N1	0.3647 (3)	0.1864 (3)	−0.12566 (17)	0.0697 (9)	
N2	0.4425 (3)	0.2831 (3)	−0.15544 (17)	0.0700 (9)	
C1	0.0865 (5)	−0.0115 (4)	0.2440 (3)	0.0860 (13)	
H1A	0.1464	0.0548	0.2679	0.103*	
H1B	0.0160	0.0232	0.2023	0.103*	
H1C	0.0455	−0.0511	0.2875	0.103*	
C2	0.2791 (4)	−0.1677 (4)	0.2699 (2)	0.0789 (12)	
H2A	0.3308	−0.2290	0.2443	0.095*	
H2B	0.3394	−0.1025	0.2952	0.095*	
H2C	0.2358	−0.2076	0.3123	0.095*	
C3	0.0725 (4)	−0.2149 (4)	0.1647 (3)	0.0812 (12)	
H3A	0.1234	−0.2768	0.1390	0.097*	
H3B	0.0313	−0.2539	0.2084	0.097*	
H3C	0.0022	−0.1801	0.1230	0.097*	
C4	0.1693 (3)	−0.1097 (3)	0.2024 (2)	0.0553 (8)	
C5	0.2384 (3)	−0.0420 (3)	0.13559 (18)	0.0466 (7)	
C6	0.2082 (4)	−0.0674 (3)	0.0497 (2)	0.0576 (9)	
H6A	0.1482	−0.1327	0.0313	0.069*	
C7	0.2645 (4)	0.0019 (3)	−0.0089 (2)	0.0584 (9)	
H7A	0.2419	−0.0171	−0.0659	0.070*	
C8	0.3550 (3)	0.0996 (3)	0.01657 (18)	0.0451 (7)	
C9	0.3904 (3)	0.1238 (3)	0.10244 (18)	0.0497 (7)	
H9A	0.4537	0.1866	0.1209	0.060*	
C10	0.3322 (3)	0.0545 (3)	0.16006 (18)	0.0515 (8)	
H10A	0.3560	0.0729	0.2170	0.062*	
C11	0.4060 (3)	0.1801 (3)	−0.04533 (19)	0.0483 (7)	
C12	0.5233 (3)	0.3273 (3)	−0.09054 (18)	0.0489 (7)	
C13	0.6256 (3)	0.4270 (3)	−0.08675 (18)	0.0467 (7)	
C14	0.7057 (3)	0.4607 (3)	−0.01123 (19)	0.0564 (8)	
H14A	0.6924	0.4211	0.0384	0.068*	
C15	0.8054 (4)	0.5533 (3)	−0.0093 (2)	0.0592 (9)	
H15A	0.8589	0.5742	0.0417	0.071*	
C16	0.8265 (3)	0.6151 (3)	−0.08216 (19)	0.0487 (7)	
C17	0.7452 (3)	0.5817 (3)	−0.1574 (2)	0.0524 (8)	
H17A	0.7576	0.6222	−0.2069	0.063*	
C18	0.6460 (3)	0.4893 (3)	−0.16024 (19)	0.0522 (8)	
H18A	0.5926	0.4685	−0.2113	0.063*	
C19	0.9346 (4)	0.7153 (3)	−0.0792 (2)	0.0638 (9)	
H19A	0.9803	0.7254	−0.0223	0.077*	
H19B	1.0000	0.6909	−0.1144	0.077*	
H19C	0.8927	0.7936	−0.0990	0.077*	
O1	0.5059 (2)	0.26623 (19)	−0.01819 (12)	0.0487 (5)	

### Atomic displacement parameters (Å^2^)

**Table:**

	*U*^11^	*U*^22^	*U*^33^	*U*^12^	*U*^13^	*U*^23^
N1	0.079 (2)	0.080 (2)	0.0463 (16)	−0.0276 (17)	−0.0004 (14)	0.0102 (14)
N2	0.079 (2)	0.078 (2)	0.0500 (16)	−0.0269 (17)	−0.0014 (14)	0.0158 (15)
C1	0.099 (3)	0.074 (3)	0.098 (3)	−0.001 (2)	0.058 (3)	0.005 (2)
C2	0.100 (3)	0.080 (3)	0.058 (2)	0.001 (2)	0.018 (2)	0.014 (2)
C3	0.095 (3)	0.078 (3)	0.075 (3)	−0.030 (2)	0.026 (2)	0.006 (2)
C4	0.065 (2)	0.0505 (18)	0.0529 (18)	−0.0047 (16)	0.0184 (16)	0.0028 (15)
C5	0.0499 (17)	0.0459 (17)	0.0452 (16)	0.0006 (14)	0.0107 (13)	0.0006 (13)
C6	0.069 (2)	0.0522 (19)	0.0529 (19)	−0.0228 (17)	0.0126 (16)	−0.0095 (15)
C7	0.074 (2)	0.058 (2)	0.0426 (17)	−0.0145 (17)	0.0085 (15)	−0.0071 (14)
C8	0.0458 (16)	0.0447 (16)	0.0448 (16)	−0.0008 (13)	0.0071 (12)	0.0006 (13)
C9	0.0479 (17)	0.0523 (18)	0.0476 (17)	−0.0092 (14)	0.0039 (13)	0.0010 (14)
C10	0.0572 (19)	0.0575 (19)	0.0382 (15)	−0.0067 (15)	0.0027 (13)	−0.0037 (14)
C11	0.0484 (17)	0.0494 (18)	0.0464 (17)	−0.0052 (14)	0.0054 (13)	0.0013 (13)
C12	0.0515 (17)	0.0518 (18)	0.0428 (16)	0.0034 (15)	0.0058 (13)	0.0090 (13)
C13	0.0454 (16)	0.0481 (17)	0.0459 (16)	0.0006 (14)	0.0048 (13)	0.0048 (13)
C14	0.064 (2)	0.063 (2)	0.0417 (16)	−0.0039 (17)	0.0091 (14)	0.0118 (15)
C15	0.066 (2)	0.062 (2)	0.0460 (18)	−0.0082 (18)	0.0000 (15)	0.0004 (15)
C16	0.0491 (17)	0.0425 (16)	0.0549 (18)	0.0034 (14)	0.0091 (14)	0.0007 (14)
C17	0.0590 (19)	0.0486 (18)	0.0507 (18)	0.0023 (15)	0.0120 (15)	0.0084 (14)
C18	0.0584 (19)	0.0527 (18)	0.0436 (16)	−0.0038 (15)	0.0021 (14)	0.0046 (14)
C19	0.064 (2)	0.057 (2)	0.071 (2)	−0.0054 (17)	0.0116 (18)	−0.0031 (17)
O1	0.0520 (12)	0.0497 (12)	0.0442 (11)	−0.0040 (10)	0.0066 (9)	0.0057 (9)

### Geometric parameters (Å, °)

**Table:**

N1—C11	1.294 (4)	C8—C9	1.395 (4)
N1—N2	1.411 (4)	C8—C11	1.462 (4)
N2—C12	1.298 (4)	C9—C10	1.379 (4)
C1—C4	1.543 (5)	C9—H9A	0.9300
C1—H1A	0.9599	C10—H10A	0.9300
C1—H1B	0.9600	C11—O1	1.365 (4)
C1—H1C	0.9601	C12—O1	1.367 (3)
C2—C4	1.536 (5)	C12—C13	1.458 (4)
C2—H2A	0.9601	C13—C14	1.388 (4)
C2—H2B	0.9600	C13—C18	1.397 (4)
C2—H2C	0.9601	C14—C15	1.389 (4)
C3—C4	1.532 (5)	C14—H14A	0.9300
C3—H3A	0.9599	C15—C16	1.389 (4)
C3—H3B	0.9599	C15—H15A	0.9300
C3—H3C	0.9601	C16—C17	1.389 (4)
C4—C5	1.540 (4)	C16—C19	1.503 (4)
C5—C6	1.394 (4)	C17—C18	1.382 (4)
C5—C10	1.396 (4)	C17—H17A	0.9300
C6—C7	1.381 (4)	C18—H18A	0.9300
C6—H6A	0.9300	C19—H19A	0.9601
C7—C8	1.388 (4)	C19—H19B	0.9599
C7—H7A	0.9302	C19—H19C	0.9599
			
C11—N1—N2	105.9 (3)	C10—C9—C8	120.2 (3)
C12—N2—N1	106.8 (3)	C10—C9—H9A	119.9
C4—C1—H1A	109.5	C8—C9—H9A	119.9
C4—C1—H1B	109.4	C9—C10—C5	122.1 (3)
H1A—C1—H1B	109.5	C9—C10—H10A	118.9
C4—C1—H1C	109.6	C5—C10—H10A	119.0
H1A—C1—H1C	109.5	N1—C11—O1	112.5 (3)
H1B—C1—H1C	109.5	N1—C11—C8	128.5 (3)
C4—C2—H2A	109.4	O1—C11—C8	118.9 (3)
C4—C2—H2B	109.4	N2—C12—O1	111.7 (3)
H2A—C2—H2B	109.5	N2—C12—C13	129.1 (3)
C4—C2—H2C	109.5	O1—C12—C13	119.3 (3)
H2A—C2—H2C	109.5	C14—C13—C18	118.7 (3)
H2B—C2—H2C	109.5	C14—C13—C12	121.2 (3)
C4—C3—H3A	109.4	C18—C13—C12	120.0 (3)
C4—C3—H3B	109.6	C13—C14—C15	120.4 (3)
H3A—C3—H3B	109.5	C13—C14—H14A	119.8
C4—C3—H3C	109.5	C15—C14—H14A	119.8
H3A—C3—H3C	109.5	C14—C15—C16	121.1 (3)
H3B—C3—H3C	109.5	C14—C15—H15A	119.4
C3—C4—C2	108.4 (3)	C16—C15—H15A	119.5
C3—C4—C5	112.5 (3)	C15—C16—C17	118.1 (3)
C2—C4—C5	109.8 (3)	C15—C16—C19	120.6 (3)
C3—C4—C1	108.9 (3)	C17—C16—C19	121.3 (3)
C2—C4—C1	109.2 (3)	C18—C17—C16	121.3 (3)
C5—C4—C1	108.2 (3)	C18—C17—H17A	119.3
C6—C5—C10	116.7 (3)	C16—C17—H17A	119.4
C6—C5—C4	123.6 (3)	C17—C18—C13	120.3 (3)
C10—C5—C4	119.6 (3)	C17—C18—H18A	119.8
C7—C6—C5	121.9 (3)	C13—C18—H18A	119.9
C7—C6—H6A	119.1	C16—C19—H19A	109.5
C5—C6—H6A	119.0	C16—C19—H19B	109.4
C6—C7—C8	120.4 (3)	H19A—C19—H19B	109.5
C6—C7—H7A	119.8	C16—C19—H19C	109.5
C8—C7—H7A	119.8	H19A—C19—H19C	109.5
C7—C8—C9	118.6 (3)	H19B—C19—H19C	109.5
C7—C8—C11	120.7 (3)	C11—O1—C12	103.2 (2)
C9—C8—C11	120.5 (3)		
			
C11—N1—N2—C12	−0.4 (4)	C9—C8—C11—O1	10.5 (4)
C3—C4—C5—C6	4.5 (5)	N1—N2—C12—O1	0.3 (4)
C2—C4—C5—C6	125.3 (4)	N1—N2—C12—C13	179.2 (3)
C1—C4—C5—C6	−115.7 (4)	N2—C12—C13—C14	−178.7 (3)
C3—C4—C5—C10	−178.5 (3)	O1—C12—C13—C14	0.1 (5)
C2—C4—C5—C10	−57.8 (4)	N2—C12—C13—C18	0.4 (5)
C1—C4—C5—C10	61.2 (4)	O1—C12—C13—C18	179.2 (3)
C10—C5—C6—C7	−2.0 (5)	C18—C13—C14—C15	−1.0 (5)
C4—C5—C6—C7	175.0 (3)	C12—C13—C14—C15	178.1 (3)
C5—C6—C7—C8	0.5 (5)	C13—C14—C15—C16	0.7 (5)
C6—C7—C8—C9	1.8 (5)	C14—C15—C16—C17	−0.1 (5)
C6—C7—C8—C11	−174.8 (3)	C14—C15—C16—C19	179.9 (3)
C7—C8—C9—C10	−2.4 (5)	C15—C16—C17—C18	−0.2 (5)
C11—C8—C9—C10	174.2 (3)	C19—C16—C17—C18	179.8 (3)
C8—C9—C10—C5	0.8 (5)	C16—C17—C18—C13	−0.1 (5)
C6—C5—C10—C9	1.4 (5)	C14—C13—C18—C17	0.7 (5)
C4—C5—C10—C9	−175.8 (3)	C12—C13—C18—C17	−178.4 (3)
N2—N1—C11—O1	0.4 (4)	N1—C11—O1—C12	−0.2 (4)
N2—N1—C11—C8	177.0 (3)	C8—C11—O1—C12	−177.2 (3)
C7—C8—C11—N1	10.5 (5)	N2—C12—O1—C11	−0.1 (3)
C9—C8—C11—N1	−166.0 (3)	C13—C12—O1—C11	−179.1 (3)
C7—C8—C11—O1	−173.0 (3)		

### Hydrogen-bond geometry (Å, °)

**Table:**

*D*—H···*A*	*D*—H	H···*A*	*D*···*A*	*D*—H···*A*
C10—H10A···N2^i^	0.93	2.59	3.456 (5)	155

Symmetry codes: (i) *x*, −*y*+1/2, *z*+1/2.
